# Localized and Sustained Delivery of Erythropoietin from PLGA Microspheres Promotes Functional Recovery and Nerve Regeneration in Peripheral Nerve Injury

**DOI:** 10.1155/2015/478103

**Published:** 2015-03-03

**Authors:** Wei Zhang, Yuan Gao, Yan Zhou, Jianheng Liu, Licheng Zhang, Anhua Long, Lihai Zhang, Peifu Tang

**Affiliations:** ^1^Department of Orthopedics, General Hospital of Chinese PLA, Beijing 100853, China; ^2^Medical Department, Affiliated Hospital of Chinese PLA General Hospital, Beijing 100048, China

## Abstract

Erythropoietin (EPO) has been demonstrated to exert neuroprotective effects on peripheral nerve injury recovery. Though daily intraperitoneal injection of EPO during a long period of time was effective, it was a tedious procedure. In addition, only limited amount of EPO could reach the injury sites by general administration, and free EPO is easily degraded *in vivo*. In this study, we encapsulated EPO in poly(lactide-co-glycolide) (PLGA) microspheres. Both *in vitro* and *in vivo* release assays showed that the EPO-PLGA microspheres allowed sustained release of EPO within a period of two weeks. After administration of such EPO-PLGA microspheres, the peripheral nerve injured rats had significantly better recovery compared with those which received daily intraperitoneal injection of EPO, empty PLGA microspheres, or saline treatments. This was supported by the functional, electrophysiological, and histological evaluations of the recovery done at week 8 postoperatively. We conclude that sustained delivery of EPO could be achieved by using EPO-PLGA microspheres, and such delivery method could further enhance the recovery function of EPO in nerve injury recovery.

## 1. Introduction

Despite the application of modern and sophisticated techniques in the treatment, peripheral nerve injury remains challenging to surgeons. Erythropoietin (EPO), which was best known as a hematopoietic cytokine, has recently been proven to have neuroprotective effects on the peripheral nerve system [1]. Although the mechanism of EPO-mediated recovery is not fully understood, it has been demonstrated that the EPO receptor (EPOR) expressed by Schwann cells was the major target for EPO in peripheral nerve injury. By recruiting *β*1 integrin to the surface of Schwann cells, EPO promotes the migration of Schwann cells and facilitates the assembly of the provisional extracellular matrix in the injured peripheral nerve and hence improves injury recovery [2].

In order to achieve the optimal effectiveness of EPO in neural repairing, the dose and duration of EPO application are of great importance. Albeit disappointing, the optimal dosage of EPO for the treatment of peripheral nerve injury is still unknown. A study in rats showed that when EPO was administered with a daily dosage of 5000 U/kg, there was a significant increase in the axon diameter, myelin thickness, and total number of nerve fibers [1]. However, no optimal duration of EPO administration was suggested in that study. It has been shown that the functional recovery of peripheral nerve was rapidly deteriorated when EPO administration was discontinued [3]. The clearance time of systematic EPO was estimated to be 4 to 10 hours in vitreous tissue [4]. Hence, continuous injection regime is usually advised for EPO application.

Recently, a procedure of sustained-release delivery of EPO was achieved by using injectable, biocompatible, and biodegradable poly(lactide-co-glycolide) (PLGA) microspheres. This procedure not only improves the patient compliance but also the therapeutic efficacy of EPO. Protein aggregation commonly occurs during the polymer-based microencapsulation process with PLGA and limits the encapsulation yield [5]. By a formulation process involving an aqueous-aqueous emulsion formed at reduced temperature, Geng and colleagues [6] were able to load EPO into PLGA microspheres with minimal protein aggregation. Later on, the protein stability during the encapsulation was further demonstrated by showing that EPO-PLGA microspheres had a prolonged protective effect on retinal ganglion cells in optic nerve crush rats [7]. Here, we evaluate whether functional recovery of peripheral nerve injury in rats could also be prompted by the use of EPO-PLGA microspheres.

## 2. Materials and Methods

### 2.1. Preparation of the EPO-PLGA Microspheres

EPO-PLGA microspheres were prepared as described [6, 8]. Briefly, a sample of lyophilized powder containing 5000 U EPO was added to 400 mg of PLGA dissolved in 4 mL anhydrous dichloromethane. The mixture was then sonicated with a CV18 3428 probe and Vibracell pump (Sonics Materials, Danbury, USA) at 50 W for 1 min. The resulting dispersion was then introduced into 40 mL precooled (4°C) aqueous solution that contained 2% w/v polyvinyl alcohol (PVA) and 5% w/v NaCl, under magnetical mixing (Sile 98-1, Shanghai Sile Co. Ltd., China) at 2000 rpm for 1 min to form embryonic composite microspheres. Then the mixture was diluted with 500 mL 1% w/v aqueous PVA solution. The microsphere aging process was done by stirring at 100 rpm for 5 h. The hardened microspheres were then rinsed 5 times with distilled water to remove PVA and NaCl, and each time the sample was centrifuged at 200 rpm for 3 min with supernatant discarded. The final EPO-PLGA microsphere was lyophilized to remove water and solvent residues prior to storage.

### 2.2. Morphology of the EPO-PLGA Microspheres

Scanning electron microscopy (SEM) was applied to observe the shape and size of the resulting EPO-PLGA microspheres. SEM was done with an FEI SIRION 200 system (FEI Co., Hillsboro, OR) at 5 KeV sputtering energy. The powder microsphere samples were attached to a metal stub using a double-sided adhesive and were exposed to gold spray under argon atmosphere for 30 sec at 120 mA.

### 2.3. Particle Size Analysis of the EPO-PLGA Microspheres

After dispersing the EPO-PLGA microspheres in an aqueous solution of 0.1% v/v Tween-20, the size distribution of the microspheres was measured by laser light scattering (Mastersizer 2000, Malvern Instruments, UK).

### 2.4. EPO Content Determination of the EPO-PLGA Microspheres

To determine the EPO content in the microspheres, 1 mg of the microspheres was taken and dissolved in 200 *μ*L dichloromethane. Then 1 mL of 10 mM PBS was added to extract the protein with vortex. After a stable dispersed phase formed, the aqueous phase was transferred to a fresh tube. The concentration of EPO was then determined using ELISA kit (R&D DEP00, USA) according to the enclosed manual.

### 2.5. Biocompatibilities of the PLGA Microspheres

The biocompatibilities of the PLGA microspheres were evaluated with L929 cell line by measuring the relative growth rate (RGR) and the cell viabilities. Briefly, cells were seeded into 96-well plate at the density of 1 × 10^4^ cells per well in Dulbecco's Minimum Essential Medium (Gibco) containing 10% fetal bovine serum (Gibco). PLGA microspheres were added into culture medium at doses of 0 (control group), 1, 5, and 10 mg/mL. After 24 h, 48 h, and 72 h cultivation, cell densities were measured with MTT [3-(4,5-dimethylthiazol-2-yl)-2,5-diphenyltetrazolium bromide] colorimetric assay. RGR was calculated as the optical density (OD) value of experimental groups over the OD value of control group. Furthermore, after 72 h cultivation, cell viabilities were assessed using Live/Dead Cell Staining Kits (Invitrogen) according to the manufacturer's instructions.

### 2.6. *In Vitro* Release of EPO from PLGA Microspheres

Microspheres (10 mg) were added to 1 mL of 10 mM PBS and incubated at 37°C with shaking in an incubator (Fuma KYC 100C, China) at 110 rpm. At each sampling time point, the sample was centrifuged at 3000 rpm for 1 min. The supernatant was collected and the same amount of 10 mM PBS was added to the sample vial. The EPO content in the supernatant was then determined using the ELISA kit. Control experiments were done with empty PLGA microspheres.

### 2.7. Sciatic Nerve Defect Animal Model

Male adult Sprague-Dawley (SD) rats weighing 200–230 g were purchased from the Experimental Animal Center, Academy of Military Medical Science (Beijing, China). And all the animal experiments were conducted with the prior approval of Animal Experimental Ethics Committee of the local institution. The animals were housed in temperature-, humidity-, and photoperiod-controlled plastic cages and allowed free access to laboratory feed and tap water.

The sciatic nerve defect was introduced to the rats using the method as described [9]. The sciatic nerve in the right hindlimb was exposed under the anesthetized condition of intramuscular Zoletil (500 *μ*g per animal) and Rompun 2% (10 *μ*L per animal). Then a complete 2 mm nerve defect was created by cutting the nerve in the central area underneath the gluteus maximus. The surgical area was then closed with nylon sutures (6-0).

The animals were divided into 4 groups: sciatic nerve defect with saline treatment (group Saline), sciatic nerve defect with daily intraperitoneal injection of 5000 U/kg EPO in 0.5 mL saline for 2 weeks (group EPO), sciatic nerve defect with single injection of EPO-PLGA microspheres (total EPO content 5000 U/kg) in the damaged area right after the defect was introduced (group PLGA/EPO), and sciatic nerve defect with single injection of the same amount of empty PLGA microspheres as in the group PLGA/EPO in the damaged area right after the defect was introduced (group PLGA).

### 2.8. *In Vivo *Efficacy of EPO by Western Blotting

At different time points after EPO delivery (0, 3, 7 and, 14 days), animals were anesthetized again by intraperitoneal injection of sodium pentobarbital (30 mg/kg body weight). Peripheral muscle samples together with nerve tissues around injury sites (also EPO microsphere injection site) were acquired. The tissue samples were lysed in lysis buffer (20 mM Tris-HCl, 5 mM EDTA, 50 mM NaCl, and 1% SDS) supplemented with proteinase inhibitors cocktail (Roche). After being homogenized with a rotor-stator homogenizer, tissue homogenates were transferred into a centrifuge tube and centrifuged at 12,000 rpm for 15 minutes at 4°C. Supernatants were then collected. Protein content was quantified with BCA Protein Assay Kit (Thermo Scientific). For western blotting analysis, 80–120 mg proteins were loaded on a 15% SDS polyacrylamide gel. After electrophoresis, proteins were transferred to a PVDF membrane (Roche). Membranes were blocked with 5% nonfat dried milk (in TBST) and incubated overnight with anti-human EPO antibodies (Cell Signaling Technology); GAPDH (Abcam) was used as internal standard. Membranes were washed three times with TBST and incubated with HRP-conjugated secondary antibodies (Santa Cruz). Protein bands were detected with enhanced chemiluminescence reagent (Applygen). ImageJ software was employed for densitometric analysis and signal intensities were standardized to its respective GAPDH.

### 2.9. Functional and Electrophysiological Evaluations

Eight weeks after surgery, all animals were evaluated by a walking track test and an electrophysiological examination as described [1]. For the walking track test, a confined walking track (7 × 50 cm^2^) was designed such that one end of the corridor was open while the other darkened. Paws of the rats were dyed with black ink and the rats were allowed to walk on the surface of one piece of white paper multiple times to obtain measurable prints. Footprint parameters of healthy and wounded feet were measured and the sciatic functional index (SFI) was calculated as described [1]. Electrophysiological examination was conducted on the right sciatic nerve in each group by using the MEB-7102 instrument (Nihon Kohden, Osaka, Japan). Motor nerve conduction velocities (MNCV) were obtained by dividing the distance between the stimulating and recording electrodes by the distal and proximal latency as described [1].

### 2.10. Histological and Immunohistological Analysis

At predetermined time points, sciatic nerves were obtained from each group and fixed in 4% paraformaldehyde. 5 *μ*m paraffin-embedded sections were prepared and used for hematoxylin-eosin (H&E) staining and immunostaining against PGP9.5 (protein gene product 9.5, Abzoom, Wuppertal, Germany) according to the standard protocols. In addition, the slices were stained with the modified Bielschowsky silver method as previously reported [1]. The stained sections were examined using light microscopy. For each section, 10 random fields (400x magnification) in an ocular grid were selected for counting myelinated axons counts. Then the axons counts per field were calculated and compared. Axon diameter and myelin thickness were measured using IPWIN60 software (Media Cybernetics, Inc.) based on the bar scale on the figures.

### 2.11. Statistical Analysis

All results are plotted as mean ± SD. Student *t*-test is applied for statistical comparisons among groups with the software SPSS 13.0 (SPSS, Chicago, USA). A *P* value < 0.05 was considered as a significant difference and *P* value < 0.01 a statistically significant difference.

## 3. Results

### 3.1. *In Vitro *Characterization of the EPO-PLGA Microspheres

**Figure 1:**
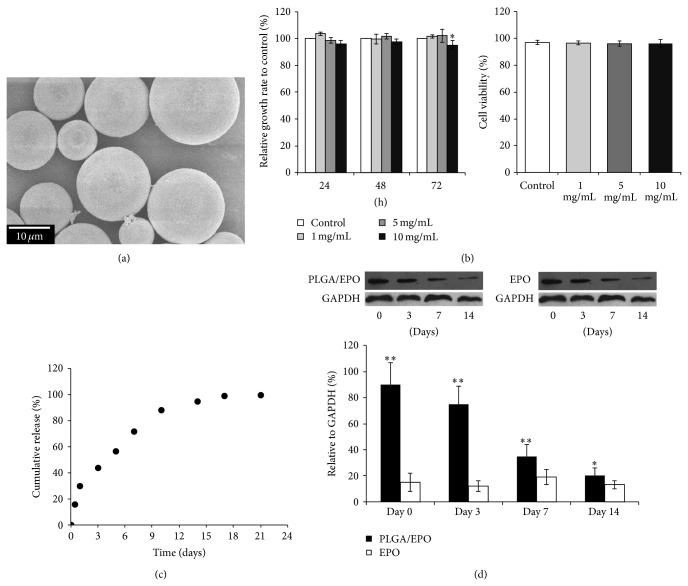
Characterization of EPO-PLGA microspheres. (a) EPO-PLGA microspheres analyzed by SEM. (b) Cell toxicity analysis of EPO-PLGA microspheres* in vitro*. Relative cell growth rates (left) and cell viabilities (right) were compared. (c)* In vitro* release profile of EPO from EPO-PLGA microspheres. Representative dot plot from triplicate experiments was shown. (d) Upper panel, western blotting analysis of EPO level from group PLGA/EPO and group EPO with GAPDH as a control. Lower panel, the relative* in vivo* concentration of EPO to the GAPDH control measured on different days after the injection was plotted and compared. Error bars stand for the S.D. from triplicate experiments (^*^ *P* < 0.05; ^**^ *P* < 0.01).

The EPO content of the formed EPO-PLGA microspheres aws determined to be 11.2 ± 2.9 U/mg by ELISA kit after being extracted from 1 mg of the microsphere samples.  Figure 1(a) shows the SEM image of EPO-PLGA microspheres. The microspheres possessed smooth surfaces and diameters ranging from 5 to 25 *μ*m. The diameter of the inner particles was less than 1/20 of that of the microspheres. Hence, the size of the EPO-PLGA microspheres met the criteria for formulating composite sustained-release polymer microspheres without causing severe burst release [10]. When treated with varying amount of such EPO-PLGA microspheres, the relative cell growth rates and cell viabilities of L929 cells had no significant changes (Figure 1(b)). This indicated that the cytotoxicity of these EPO-PLGA microspheres was minimum. The* in vitro* release profile of EPO, shown in Figure 1(c), revealed that only less than 20% of the total EPO loadings were initially burst-released at day 1 and above 90% of loadings were sustainably released up to 10 days.

### 3.2. *In Vivo* Efficacy of the EPO-PLGA Microspheres

*In vivo* efficacy of EPO from the PLGA/EPO group and EPO group was determined with western blotting analyses and compared, as shown in Figure 1(d) (upper panels). When EPO was daily injected to the rats in the EPO group with a dose of 5000 U/kg, the* in vivo* level of EPO was kept below 20% relative to the level of GAPDH which was used as a control in the western blotting assay. On the other hand, when EPO was injected in forming EPO-PLGA microspheres with a dose of 5000 U/kg (body weight of animal) in the PLGA/EPO group, its* in vivo* concentration was much higher in the earlier period after day 0. The release of EPO by the PLGA microspheres could even last until day 14 while still keeping the EPO level higher than the daily injected level in the EPO group. This demonstrated the feasibility of the sustained-release EPO-PLGA microspheres formulation in achieving* in vivo* long action by a single dose.

### 3.3. Functional and Electrophysiological Evaluations

**Figure 2:**
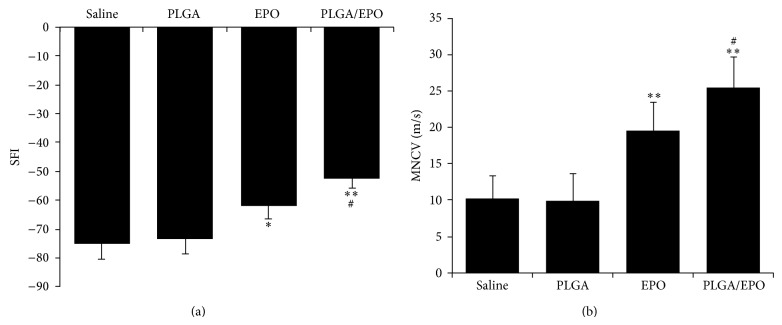
Functional and electrophysiological evaluations 8 weeks postoperatively. (a) Comparison of the mean functional recovery of each group in terms of SFI derived from walking track prints. (b) Effect of different treatments on MNCV. Data are the mean ± SD. ^*^ *P* < 0.05 and ^**^ *P* < 0.01 when compared with saline or PLGA group and ^#^ *P* < 0.05 when compared with EPO group.

Eight weeks postoperatively, the SFI values in rats in all the three groups were measured (Figure 2(a)). From the SFI values, both of the EPO and PLGA/EPO groups showed significantly better recovery than the saline and PLGA group. Furthermore, the SFI values in the PLGA/EPO group were significantly greater than those in the EPO group, indicating that the sustained release of EPO by the PLGA microspheres prompted the recovery of peripheral nerved defect in rats compared with daily injection of EPO protein.

Meanwhile, MNCV was measured to confirm the improved recovery-prompting effect of EPO by the sustained release from PLGA microspheres (Figure 2(b)). After 8 weeks of treatment, the MNCV was 19.2 ± 4.3 m/s for the EPO group, which was significantly higher than that of the untreated saline group (10.0 ± 3.8 m/s) and the PLGA group (9.5 ± 4.1 m/s). The MNCV for the PLGA/EPO group, 25.3 ± 4.6 m/s, was significantly higher compared with the EPO group.

### 3.4. Histological and Immunohistological Evaluation of the Peripheral Nerve Recovery

**Figure 3:**
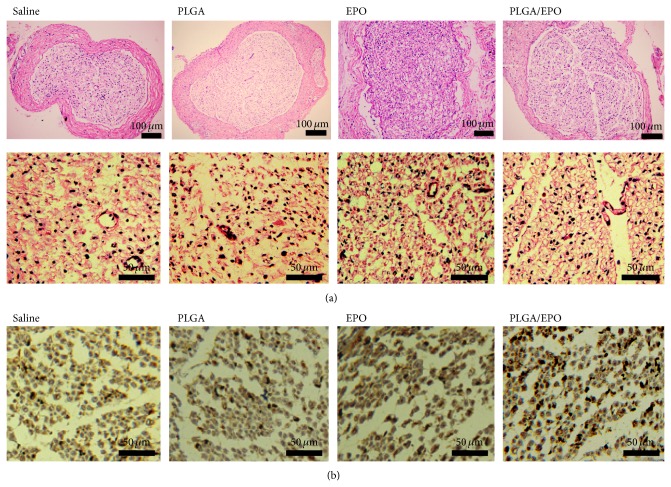
Histological and immunohistological analyses. (a) 8 weeks after surgery, the thinnest bands of scar tissue of H&E staining of sciatic nerve tissues and more regular bundles were demonstrated in animals treated with PLGA/EPO compared with saline, PLGA, and EPO treatment. (b) Immunostaining against PGP 9.5 showed that immunopositive nerve fibers or deeper stained fibers were obviously more in PLGA/EPO treated animals.

At 8 weeks after surgery, sciatic nerve tissues of injury were acquired and observed under microscope. As shown in Figure 3(a), the thinnest bands of scar tissue were found in nerves from the animals in the PLGA/EPO group. Animals receiving saline or empty PLGA microspheres treatment demonstrated severe injury of circular bundles in sciatic nerve tissues since most bundles were observed as incomplete. In animals treated by daily intraperitoneal injection of EPO, more circular bundles could be observed, indicating significant recovery of injured bundles compared with saline-treated ones. In comparison with saline and EPO treatment, sustained delivering of EPO by PLGA microspheres demonstrated the most significant improvement in recovery of injured circular bundles. As shown in Figure 3(a), lots of mature bundles with circular morphologies could be observed, which were more similar to normal nerves.

PGP 9.5 is one of the earliest neuron-specific genes to be expressed. Generally, the expression pattern of PGP 9.5 closely matches the degree of maturity of regenerated nerve fibers. Thus, we further evaluated the PGP 9.5 expression in the sciatic nerve from different groups. As shown in Figure 3(b), the intensity of immunoreactivity in the sciatic nerve from PLGA/EPO treated animal was significantly higher than those from saline, empty PLGA, and free EPO treated animals. That is, EPO treatment significantly enhanced the expression of PGP 9.5 in the regenerated sciatic nerve, and locally controlled release by PLGA significantly reinforced the therapeutic effects of EPO.

**Figure 4:**
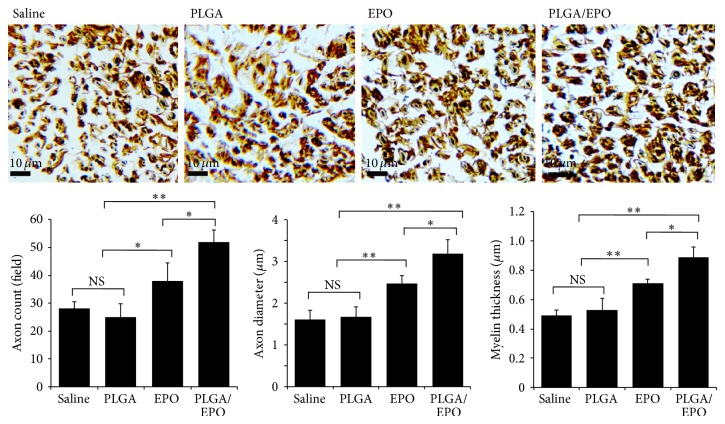
Modified Bielschowsky silver staining. 8 weeks after surgery, sciatic nerve tissues were stained with modified Bielschowsky silver method. The axon density, axon diameter, and myelin thickness were determined and compared between different groups (NS indicates not significant; ∗ indicates *P* < 0.05; ∗∗ indicates *P* < 0.01).

In addition, tissue sample from the PLGA/EPO group also showed significantly increased myelinated axons counts, higher axon diameter, and higher myelin thickness compared with the EPO group, the saline group, and the PLGA group (Figure 4). All these data together indicated strongly that the sustained EPO release achieved by the EPO-PLGA microspheres could prompt the recovery of peripheral nerve injury in rats.

## 4. Discussion

There is accumulated evidence showing that EPO has neurotrophic and neuroprotective effects not only on the central nerve system [11] but also on the peripheral nerve fibers [12–14]. One recent study showed that EPO could promote the functional recovery and enhance nerve regeneration after peripheral nerve injury in rats [1]. However, to achieve this effect on the peripheral nerve recovery, daily intraperitoneal injections of EPO should be received along a very long period due to the short lifetime of EPO in the tissue [4]. Hence, a sustained delivery of EPO would benefit the treatment technically and economically.

In this study, we encapsulate the EPO protein with PLGA microspheres [6, 8]. The biocompatibilities of such microspheres were demonstrated with the findings of no obvious cytotoxicity* in vitro* (Figure 1(b)). Both* in vitro* and* in vivo* releasing of EPO from the EPO-PLGA microspheres demonstrated sustained EPO release profiles along a period of two weeks (Figures 1(c) and 1(d)). The effect of such sustained delivery of EPO on peripheral nerve injury recovery was then studied in rat model [9]. Treatments with daily intraperitoneal injections of saline, empty PLGA microspheres, and EPO were done for comparisons. The recovery levels in each treatment group were compared 8 weeks postoperatively. Functional, electrophysiological, and histological evaluations of the recovery were done for the animals in each group.

As expected, we were able to reproduce the improvement effect on the recovery by daily intraperitoneal injections of EPO with a dosage of 5000 U/kg [1]. Compared with those of saline group and PLGA group, tissue samples from the EPO group showed more myelinated axons counts, higher axon diameter, and higher myelin thickness (Figure 4). The ~2-fold increase in MNCV of the EPO group compared to the saline or PLGA group and the improved SFI values demonstrated the recovery function of EPO in peripheral nerve injury (Figure 2). Remarkably, a significant improvement of the recovery function of EPO was achieved with our EPO-PLGA microspheres, as demonstrated in all these evaluation results. The MNCV was statistically significantly increased by ~30% compared with that of the EPO group (Figure 2(b)). The band of scar tissue was the thinnest in nerves from the animals in the PLGA/EPO group (Figure 3(a)) and also indicated the best recovery of peripheral nerve injury among all the studied groups.

The dose and duration of EPO application are essential for the recovery function. So far, no optimal dose and duration of EPO administration were suggested in the literature. Our way of sustained release of EPO by the EPO-PLGA microspheres could help in circumventing the short clearance time of systematic EPO [4]. The sustained release of EPO from the EPO-PLGA microspheres would allow better-controlled delivery of EPO to the injury site. This might facilitate the study of the optimal dose and duration of EPO administration in the treatment of peripheral nerve injury.

## 5. Conclusion

In this work, by encapsulating EPO in PLGA microspheres, we developed a localized and sustained delivery method for EPO. We demonstrated the improvement of the functional recovery effect of EPO via this delivery method in peripheral nerve injured rats model. Such EPO-PLGA microspheres have great potential in clinical treatments of peripheral nerve injuries.
