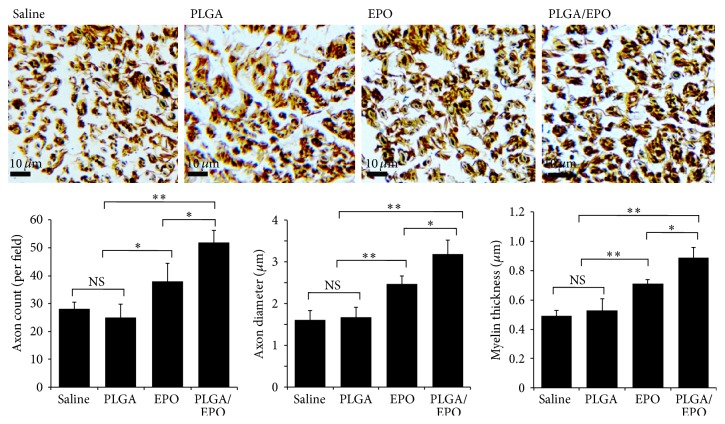# Erratum to “Localized and Sustained Delivery of Erythropoietin from PLGA Microspheres Promotes Functional Recovery and Nerve Regeneration in Peripheral Nerve Injury”

**DOI:** 10.1155/2015/214703

**Published:** 2015-07-28

**Authors:** Wei Zhang, Yuan Gao, Yan Zhou, Jianheng Liu, Licheng Zhang, Anhua Long, Lihai Zhang, Peifu Tang

**Affiliations:** ^1^Department of Orthopedics, General Hospital of Chinese PLA, Beijing 100853, China; ^2^Medical Department, Affiliated Hospital of Chinese PLA General Hospital, Beijing 100048, China

In the paper titled “Localized and Sustained Delivery of Erythropoietin from PLGA Microspheres Promotes Functional Recovery and Nerve Regeneration in Peripheral Nerve Injury” we have found that, in Figure 1(d), one panel is mistakenly a duplication of the adjacent panel. The upper two western blotting results are identical. According to our inspection of the original data sets, this is due to a mistake during the editorial process of the figures. We show here the amended Figure 1(d), with the correct image for the western blotting results of group EPO.

Due to the same reason, another mistake was made in the lower left panel of Figure 4. The label of the *y*-axis, “Axon count (field),” should read “Axon count (per field).” And a typo was found in the beginning of the second line under Section 3.1 that “aws” should read “was.”

## Figures and Tables

**Figure 1:**
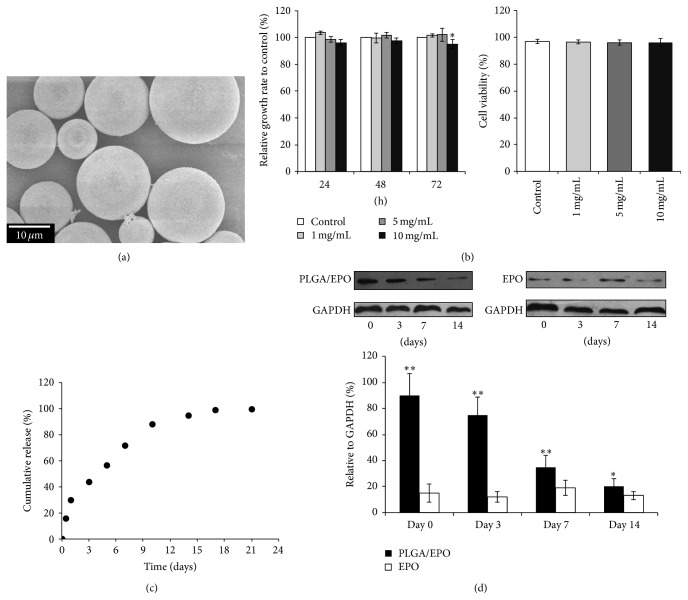


**Figure 4:**